# Supplementary material for: A systematic scoping review of health-promoting interventions for contact centre employees examined through a behaviour change wheel lens
Source: PLoS One. 2024 Mar 8;19(3):e0298150. doi: 10.1371/journal.pone.0298150 (PMC10923409; doi:10.1371/journal.pone.0298150)
Supplement: S2 File — (PDF) [file pone.0298150.s002.pdf]

Supplementary 2 - Search Strategies

<u>MEDLINE Search Strategy</u>

1. “call agent*” OR “call cent* agent*” OR “call cent* employee*”
2. Health OR “health and safety” OR “occupational health” OR “wellbeing” OR “stress” OR “workplace stress” OR “occupational stress” OR “job stress” OR “occupational ill-health” OR “job-related strain” OR “mental health” OR “physical activity” OR “sedentary behaviour” OR diet OR “healthy eating” OR smoking OR alcohol OR “working conditions” OR “work environment” OR “work organi?ation” OR “health promotion” OR “workplace solutions” OR “workplace health promotion” OR ergonomics OR “job redesign” OR “work design” OR intervention OR “quasi-experiment” OR experimental OR randomi?ed OR random* OR trial OR strateg* OR guid*
3. “contact cent*” OR “call cent*”
4. 1 AND 2
5. 4 AND 3

<u>CINAHL Search Strategy</u>

1. “Call agent*” OR “call cent* agent*” OR “call cent* employee*”

2. Health OR “health and safety” OR “occupational health” OR “wellbeing” OR “stress” OR “workplace stress” OR “occupational stress” OR “job stress” OR “occupational ill-health” OR “job-related strain” OR “mental health” OR “physical activity” OR “sedentary behaviour” OR diet OR “healthy eating” OR smoking OR alcohol OR “working conditions” OR “work environment” OR “work organi?ation” OR “health promotion” OR “workplace solutions” OR “workplace health promotion” OR ergonomics OR “job redesign” OR “work design” OR intervention OR “quasi-experiment” OR experimental OR randomi?ed OR random* OR trial OR strateg* OR guid*

3. “contact cent*” OR “call cent*”

4. 1 AND 2

5. 4 AND 3

<u>PsycInfo Search Strategy</u>

1. “Call agent*” OR “call cent* agent*” OR “call cent* employee*”

2. Health OR “health and safety” OR “occupational health” OR “wellbeing” OR “stress” OR “workplace stress” OR “occupational stress” OR “job stress” OR “occupational ill-health” OR “job-related strain” OR “mental health” OR “physical activity” OR “sedentary behaviour” OR diet OR “healthy eating” OR smoking OR alcohol OR “working conditions” OR “work environment” OR “work organi?ation” OR “health promotion” OR “workplace solutions” OR “workplace health promotion” OR ergonomics OR “job redesign” OR “work design” OR intervention OR “quasi-experiment” OR experimental OR randomi?ed OR random* OR trial OR strateg* OR guid*

3. “contact cent*” OR “call cent*”

4. 1 AND 2

5. 4 AND 3

Web of Science Search Strategy

1. “call agent*” OR “contact cent* agent*” OR “call cent* agent” OR “call cent* employee*”
2. Health OR “health and safety” OR “occupational health” OR “wellbeing” OR “stress” OR “workplace stress” OR “occupational stress” OR “job stress” OR “occupational ill-health” OR “job-related strain” OR “mental health” OR “physical activity” OR “sedentary behaviour” OR diet OR “healthy eating” OR smoking OR alcohol OR “working conditions” OR “work environment” OR “work organi?ation” OR “health promotion” OR “workplace solutions” OR “workplace health promotion” OR ergonomics OR “job redesign” OR “work design” OR intervention OR “quasi-experiment” OR “experimental” OR “randomi?ed” OR trial OR “random*” OR strateg* OR guid*
3. “contact cent*” OR “call cent*” OR “service cent*”
4. 1 AND 2
5. 4 AND 3
