# Supplementary material for: A systematic scoping review of health-promoting interventions for contact centre employees examined through a behaviour change wheel lens
Source: PLoS One. 2024 Mar 8;19(3):e0298150. doi: 10.1371/journal.pone.0298150 (PMC10923409; doi:10.1371/journal.pone.0298150)
Supplement: S3 File — (PDF) [file pone.0298150.s003.pdf]

S3 File – Data Extraction Tool

Evidence Source Details and Characteristics

Citation details e.g. author/s, year, title, journal/company, URL

Details/Results Extracted from Source of Evidence

Country

Setting (available details of contact centre e.g. organisational level/structure/ type)

Participants details e.g. number, details (eligibility), demographics

Source purpose and aims (broad)

Methodology design/ Recruitment design/ Data analysis

Key characteristics of health intervention/ policy document

Authors' conclusions (Effectiveness? Feasibility? Acceptability? Recommendation?)
