# Supplementary material for: A systematic scoping review of health-promoting interventions for contact centre employees examined through a behaviour change wheel lens
Source: PLoS One. 2024 Mar 8;19(3):e0298150. doi: 10.1371/journal.pone.0298150 (PMC10923409; doi:10.1371/journal.pone.0298150)
Supplement: S4 File — (PDF) [file pone.0298150.s004.pdf]

S4 File: Reference list for the 26 sources (28 intervention studies) identified

1. Allexandre D, Bernstein AM, Walker E, Hunter J, Roizen MF, Morledge TJ. A Web-Based Mindfulness Stress Management Program in a Corporate Call Center: A Randomized Clinical Trial to Evaluate the Added Benefit of Onsite Group Support. J Occup Environ Med. 2016;58(3):254-64.

2. Bond FW, Flaxman PE, Bunce D. The influence of psychological flexibility on work redesign: mediated moderation of a work reorganization intervention. J Appl Psychol. 2008;93(3):645-54.

3. Chau JY, Sukala W, Fedel K, Do A, Engelen L, Kingham M, et al. More standing and just as productive: Effects of a sit-stand desk intervention on call center workers' sitting, standing, and productivity at work in the Opt to Stand pilot study. Prev Med Rep. 2016;3:68-74.

4. Chi CF, Lin YH. Effects of using a screen filter on call center workers' visual fatigue measurement. Percept Mot Skills. 2009;108(1):229-38.

5. Cook C, Burgess-Limerick R. The effect of forearm support on musculoskeletal discomfort during call centre work. Appl Ergon. 2004;35(4):337-42.

6. Garrett G, Benden M, Mehta R, Pickens A, Peres SC, Zhao H. Call Center Productivity Over 6 Months Following a Standing Desk Intervention. IIE Transactions on Occupational Ergonomics and Human Factors. 2016;4(2-3):188-95.

7. Holman D, Axtell C. Can job redesign interventions influence a broad range of employee outcomes by changing multiple job characteristics? A quasi-experimental study. J Occup Health Psychol. 2016;21(3):284-95.

8. Holman D, Axtell C, Sprigg C, Totterdell P, Wall T. The mediating role of job characteristics in job redesign interventions: A serendipitous quasi-experiment. Journal of Organizational Behavior. 2009;31:84-105.

9. Kennedy JJ, Pretorius M. Integrating a portable biofeedback device into call centre environments to reduce employee stress: Results from two pilot studies. Journal of Workplace Behavioral Health. 2008;23(3):295-307.

10. Kirk E, Strong J, Burgess-Limerick R. Developing computer competencies for eWorkers within call centres. Work. 2013;46(3):283-95.

11. Krajewski J, Sauerland M, Wieland R. Relaxation-induced cortisol changes within lunch breaks–an experimental longitudinal worksite field study. Journal of Occupational and Organizational Psychology. 2011;84(2):382-94.

12. Krajewski J, Wieland R, Sauerland M. Regulating strain states by using the recovery potential of lunch breaks. J Occup Health Psychol. 2010;15(2):131-9.

13. Lehto L, Rantala L, Vilkman E, Alku P, Bäckström T. Experiences of a short vocal training course for call-centre customer service advisors. Folia Phoniatr Logop. 2003;55(4):163-76.

14. Mishra GA, Majmudar PV, Gupta SD, Rane PS, Hardikar NM, Shastri SS. Call centre employees and tobacco dependence: making a difference. Indian J Cancer. 2010;47 Suppl 1:43-52.

15. Morris AS, Murphy RC, Hopkins ND, Low DA, Healy GN, Edwardson CL, et al. Sit Less and Move More-A Multicomponent Intervention With and Without Height-Adjustable Workstations in Contact Center Call Agents: A Pilot Randomized Controlled Trial. J Occup Environ Med. 2021;63(1):44-56.

16. Morris AS, Murphy RC, Shepherd SO, Healy GN, Edwardson CL, Graves LEF. A multi-component intervention to sit less and move more in a contact centre setting: a feasibility study. BMC Public Health. 2019;19(1):292.

17. Pickens AW, Kress MM, Benden ME, Zhao H, Wendel M, Congleton JJ. Stand-capable desk use in a call center: a six-month follow-up pilot study. Public Health. 2016;135:131-4.

18. Rempel DM, Krause N, Goldberg R, Benner D, Hudes M, Goldner GU. A randomised controlled trial evaluating the effects of two workstation interventions on upper body pain and incident musculoskeletal disorders among computer operators. Occup Environ Med. 2006;63(5):300-6.

19. Schneider-Stickler B, Knell C, Aichstill B, Jocher W. Biofeedback on voice use in call center agents in order to prevent occupational voice disorders. J Voice. 2012;26(1):51-62.

20. Sharifi AS, Danesh MK, Gholamnia R. Improvements in musculoskeletal symptoms, mental workload and mental fatigue: Effects of a multicomponent ergonomic intervention among call center workers. Work. 2022;72(2):765-74.

21. Tham KW. Effects of temperature and outdoor air supply rate on the performance of call center operators in the tropics. Indoor Air. 2004;14 Suppl 7:119-25.

22. Thatcher A, Adamson K, Bloch LG, Kalantzis A. Do indoor plants improve performance and well-being in offices? Divergent results from laboratory and field studies. Journal of Environmental Psychology. 2020;71:101487.

23. Wargocki P, Wyon DP, Fanger PO. The performance and subjective responses of call-center operators with new and used supply air filters at two outdoor air supply rates. Indoor Air. 2004;14 Suppl 8:7-16.

24. Workman M. Results from organizational development interventions in a technology call center. Human Resource Development Quarterly. 2003;14(2):215-30.

25. Workman M, Bommer W. Redesigning Computer Call Center Work: A Longitudinal Field Experiment. Journal of Organizational Behavior. 2004;25(3):317-37.

26. Yeşilyurt M, Yelken K. The Efficacy of Voice Therapy in Call Center Agents with Disphonia. Journal of Academic Research in Medicine. 2020;10:185-8.
