# Supplementary material for: A systematic scoping review of health-promoting interventions for contact centre employees examined through a behaviour change wheel lens
Source: PLoS One. 2024 Mar 8;19(3):e0298150. doi: 10.1371/journal.pone.0298150 (PMC10923409; doi:10.1371/journal.pone.0298150)
Supplement: S1 Table — (PDF) [file pone.0298150.s005.pdf]

**S1 Table – Intervention Description Table**

| Intervention description table for the 28 included intervention studies (from 26 intervention articles) | | |
|---|---|---|
| **Authors (Year)** | **Intervention** | **Intervention description** |
| Allexandre et al (2016) **[1]** | Web-Based Mindfulness Stress Management Program (WSM) and group support | **Web-Based Mindfulness Stress Management Program:** an 8-week online, interactive, educational program based on mindfulness mediation. Participants receive different themes each week through audio (online or downloaded) to be played at home or in work. There are also educational articles and (e.g., mindfulness in everyday eating) x2 email reminders. **Group support**: groups of 11-12 for 1 hour (once a week) for 8 weeks in work time (during low call volume periods). They started with a deep breathing exercise for 2 minutes, then listened to a 10-minute audio recording of the weekly lesson and practiced the 20 to 30 min guided meditation exercise of the week. The final 20 minutes was a discussion, sharing of positive experiences. **Clinical support**: followed the same meeting schedule as groups, but their discussions were facilitated by a clinical counsellor/ social worker. |
| Bond, Flaxman & Blunce (2008) **[2]** | Participatory Action Research (PAR) Intervention | **Procedures**: Formation of a steering committee (12 team members); research team facilitated two. 2-hour steering committee meetings began two months after the Time 1 questionnaires. Meeting 1- committee provided with results from Time 1 that identified work organisation characteristics. **Committee aims were to**: (1) identify specific instances of these problematic aspects of work organisation and (2) recommend changes that might address these problems to improve the outcomes. Committee consulted with colleagues between meetings to finalise recommendations for change. **Committee proposed**: team members be given greater control and influence over their team's daily and weekly work plans, and be allowed more discretion over the selection, timing and ordering of their work tasks. They implemented systems for this during the 5th month, allowing members to participate in work planning process. They also implemented regular one-to-one meetings with team leaders to solve problems and develop/plan training needs. |
| Chau et al (2015) **[3]** | Sit-stand desk | Participants received a sit-stand desk (Rumba "2 Stage" Sit-Stand Workstation from Zenith) and brief training on its use. Daily e-mail reminders to stand up more during the workday were sent out for the first 2 weeks after sit-stand desks were installed. |

| | | |
|---|---|---|
| Chi & Lin (2009)<br>**[4]** | Screen filter | The manufacturer of the screen filter claimed its product has ergonomic advantages for users, such as reduction of surface reflection and glare up to 98%, with 90% high transmission to maintain the brightness and colour presence of the LCD screen. |
| Cook et al (2004)<br>**[5]** | Forearm support | Workstations were adjusted to support forearms (but not elbows) on the desk surface, maintaining neutral shoulder elevation. The keyboard was positioned so that the top row of keys was level with fingertips when the forearms were supported comfortably on the worksurface. The mouse was positioned next to the keyboard, so that at least half of the forearm was supported on the desk while working. Participants were also given a prompt sheet outlining how to maintain the forearm support position. |
| Garrett et al (2016)<br>**[6]** | Stand-capable desks | Employees received brief training on their new workstations. The workstations used for this intervention were the SteelCase™ (Grand Rapids, MI) Series 5 Desk (sit-to-stand) which were adjustable with an electronic motor from 65 cm to 130 cm, allowing the user to adjust the desk surface height for both sitting and standing. In comparison to a sit-to-stand workstation that can be adjusted by the user to any posture between seated and standing during the day, the stand-biased workstation is adjusted to a range of standing heights. The stand-biased workstations had a raised height or bar height task chair. The Neutral Posture Inc. (Bryan, TX, USA) U4IA4692 Mesh Back Stool was used, with attached foot platform at 15.24 and 25.4 cm and a seat height that can be adjusted between 64.77 and 91.44 cm (Fig. 2). Footrests that allow a user to prop one foot up at 20.32 or 30.48 cm were purchased for stand-biased desk users. Anti-fatigue mats were purchased for sit-to-stand users. Monitor arms for a dual monitor set-up were purchased and installed at each workstation. |
| Holman et al (2009)<br>**[7]** | Participatory job redesign intervention | The job redesign process had two main phases: 1) Assessment and redesign, and 2) implementation. **Assessment and redesign:** Teams identifying core job tasks and obstacles that prevent effective working. The current job design was then rated (scale 1-10) for effects on wellbeing and performance. The job characteristics were *job control, skill utilization, feedback, participation,* and *task obstacles*. Teams then discussed job characteristics that would maximise wellbeing and performance. **Implementation**: Teams given responsibility to implement the proposed job redesign changes. Two representatives per team agreed to monitor progress on job design changes, and to attend three implementation meetings (spread over 3 |

| | | |
|---|---|---|
| | | months) with the research team to discuss progress. *Job control* - the adoption of new tasks and procedures e.g. changing customers names and access to new customer info; *Participation* - involvement in design of new IT system, team member setting work schedules and breaks; *Skill utilization* - training on the new tasks previously outlined; *Feedback* - performance criteria specified more clearly and feedback given more often; *removal of task obstacles* - visiting other teams to increase knowledge share and understand other department procedures. |
| Holman & Axtell (2016) **[8]** | Participatory job redesign intervention | The job redesign process had two main phases: 1) Assessment and redesign, and 2) implementation. Employees participated in the **assessment phases** (2-day workshop) facilitated by the research team; this included the discussion of advisor survey results. Participants proposed changes perceived to have a positive impact on well-being and performance – these were discussed between employees, management and researchers. **Implementation**: advisors were given responsibility for a range of administration tasks (previously conducted by team leaders) such as organising breaks, logging working time and performance data. Advisors were also given greater discretion over handling 'minor' queries/complaints and trained on this. Agents and team leaders improved the clarity of the performance criteria and simplified the feedback process. Advisors were given responsibility for running and delivering weekly team briefing sessions. **Teams were tasked with implementing the proposed initiatives** within 4 months and monitoring the effectiveness with the support of the research team. |
| Kennedy & Pretorius (2008) **[9]** | Portable heart rate variability (HRV) biofeedback device | A biofeedback device named the 'StessEraser'. The device guides users to maximise their HRV by finding their unique breathing pattern via their heart rate wave so that respiration and heart rate (HR) covary in a synchronous phase relationship. This usually involves breathing somewhere between 4.5 and 7.5 breaths per minute but varies from person to person. Each time users meet a certain threshold, they receive points. Points are awarded for smooth waves when HRV is increased but not awarded when disruptions in the wave occur through improper breathing or excessive limbic activity. |
| Kirk et al (2013) **[10]** | Office ergonomic checklist *(Study 1)* | Office ergonomic checklist designed by the in-house occupational health and safety officer. The paper-based checklist outlined office ergonomic recommendations for the placement and/or adjustment of equipment and furniture at static computer workstations. |

| | | |
|---|---|---|
| | Skill-based training programme to self-manage Work-Related Musculo-Skeletal Disorder risk factors *(Study 2)* | One-on-one skill-based ergonomic intervention delivered in-situ by the researcher. Designed around a series of actions, training demonstrated how to judge the 'best possible' position of furniture and equipment and how to make those adjustments. The training sequence coincided with an operator stepping up to, sitting down at the workstation, and getting ready to start work. Delivery involved demonstration, followed by the trainee rehearsing the skills for each ergonomic recommendation. Adjustments were made based on the operators' or trainees' personal anthropometry, allowing recommendations to be fine-tuned to meet individual needs. Training actions were supported by an explanation of the effect of this ergonomic recommendation on work posture, the benefits of achieving a relaxed neutral work position, and the health consequences of common workstation adjustment errors. Training concluded with instructions that made trainees consciously aware of the relaxed neutral work posture achieved through the process of making the ergonomic adjustments, the need to actively 'rest' this position at the end of each call and were asked for feedback on how this work posture felt. |
| Krajewsji, Wieland & Sauerland (2010) **[11]** | Progressive muscle relaxation (PMR) break in a 'silent room' | During the first part of the lunch break (12:00 to 12:20) a snack was served. The second part of the break (12:30 –13:00) took place in a noise-subdued, dimly lit (10 lux), gaze-dense lockable cabin, called the "silent room," wearing eye masks. PMR instructions were given via wireless headphones (including calm instrumental background music) while the subjects lay on medical daybeds. |
| Krajewski, Sauerland & Rainer (2011) **[12]** | Progressive muscle relaxation (PMR) break in a 'silent room' | During the first part of the lunch break (12:00 to 12:20) a snack was served. The second part of the break (12:30 –13:00) took place in a noise-subdued, dimly lit (10 lux), gaze-dense lockable cabin, called the "silent room," wearing eye masks. PMR instructions were given via wireless headphones (including calm instrumental background music) while the subjects lay on medical daybeds. |
| Lehto et al (2003) **[13]** | 2-day vocal training course | Vocal training for 2 days by a speech-language therapist, including both indirect and direct therapy methods. The first day consisted of 6 h divided into two sections. The first section consisted of lectures on the theory of voice production, resonance and articulation. The basics of vocal hygiene, balanced breathing patterns and the importance of good body posture were also discussed as tools to reduce tension when speaking. The subjects were also provided with information about the kinds of foods and drinks that may have a negative effect on their voice. The second part |

| | | |
|---|---|---|
| | | of the day included vocal activities. The subjects were taught different vocal exercises: they were informed of how to use their voice more economically and they learned exercises to warm up or cool down their voice. The exercises that were used are widely recognised and clinically used. The whole second day of the training course was spent practising these vocal exercises. |
| Mishra et al (2010) **[14]** | Health Education sessions and focus group | The interactive Health education session covered current statistics of tobacco use, health hazards of tobacco, the different methods of quitting tobacco, and information about the trial. The entire management and all the employees (whether they were tobacco users or not) were invited to participate in the health awareness lectures.<br><br>The focus groups were conducted in small groups of 7-10 employees. The participants were employees consuming tobacco. These interactive counselling sessions were conducted by an expert tobacco counsellor. The initial sessions were aimed at initiating the thought process among tobacco users regarding positive need to quit tobacco, make them reflect on their own strengths and coping capacity, how they can use the same regarding tobacco cessation, and to promote decisions toward healthy lifestyles.<br><br>Subsequent sessions focused on sharing of quitting experiences by the tobacco users and coping with withdrawals. Later sessions focused on how to prevent relapses and need to maintain sustained efforts at quitting. |
| | Health Education sessions followed by focus group and Behavioural therapy (one-to-one counselling) | In addition to education sessions and focus groups, one-to-one counselling was provided to the tobacco users in the third arm. This involved added resources in the form of separate time dedicated to each tobacco user. The rationalisations for continuing tobacco use at the individual level were addressed. |
| | Health Education sessions followed by focus group, Behavioural therapy (one-to-one counselling), and Pharmacotherapy | In addition to education sessions, focus groups and behavioural therapy this intervention also added Pharmacotherapy in the form of bupropion. This was offered to tobacco users based on the individual need assessment. This was offered in the preparatory phase. |

| | | |
|---|---|---|
| Morris et al (2021) **[15]** | Multicomponent intervention with height-adjustable workstations (SLAMM+) | A height-adjustable workstation (Posturite DeskRite 100 or VARIDESK ProPlus) was installed outside of work hours. This allowed work to be conducted in either a seated or a standing posture and enabled frequent transitions between postures. Attached were instructions. **Interpersonal strategies:** Stand Up Champions and team leaders were used to encourage and support participants to sit less and move more at work through discussions and modelling; there was no pressure of coercion. Support emails were sent in months 1-3, and monthly in months 4-10. **Intrapersonal Strategies:** 4 30-min researcher-led education and training sessions in working hours. Sessions outlined, and reinforced (weeks 3, 9, month 6) the intervention aims and benefits of sitting less and moving more and identified opportunities and strategies for this with emphasis on frequent posture changes, active breaks and standing work (SLAMM+ only). In week 1, agents worked collectively to identify practical ways to incorporate sitting less and moving more into their working practice. The sessions also introduced (week 1) and reinforced (week 3 and 9) a goal setting and self-monitoring strategy to gradually increase standing and light activity (walking) at work to 2-4 h/day. Agents received a diary and timer and were encouraged to monitor (timer) and log (diary) their daily standing (weeks 1-12) and walking (weeks 4-12) time at work against incremental goals suggested in the diary. Agents received paper-based individual feedback, and group-level feedback via presentations, on anthropometric, cardiometabolic (both week 1, month 6) and behavioural outcomes (week 9, month 6). |
| | Multicomponent intervention without height-adjustable workstations (SLAMM) | **Interpersonal strategies:** Stand Up Champions and team leaders were to encourage and support participants to sit less and move more at work through discussions and modelling; there was no pressure of coercion. **Intrapersonal Strategies:** In week 1, agents worked collectively to identify practical ways to incorporate sitting less and moving more into their working practice. The sessions also introduced (week 1) and reinforced (week 3 and 9) a goal setting and self-monitoring strategy to gradually increase standing and light activity (walking) at work to 2-4 h/day. Agents received a diary and timer and were encouraged to monitor (timer) and log (diary) their daily standing (weeks 1-12) and walking (weeks 4-12) time at work against incremental goals suggested in the diary. Agents received paper-based individual feedback, and group-level feedback via |

| | | |
|---|---|---|
| | | presentations, on anthropometric, cardiometabolic (both week 1, month 6) and behavioural outcomes (week 9, month 6). |
| Morris et al (2019) **[16]** | Multicomponent intervention with height-adjustable workstations | Height-adjustable workstation (Posturite DeskRite 10 small, UK) installed during work hours. Workstations allowed work to be conducted in either a seated or a standing posture and enabled frequent transitions between postures. Attached were instructions. **Interpersonal strategies:** Team leaders were specifically educated, trained and encouraged to a) encourage walking in their one-to-one and team meetings with agents, b) discuss agent experiences of the intervention during one-to-one and team meetings, c) provide daily verbal support and encouragement to agents to sit less and move more, and d) forward a weekly intervention email to their agents. The movement champion was specifically encouraged to provide daily verbal support for agents to sit less and move more, and encourage team leaders to complete the above actions. Team leaders and the movement champion left the session with a laminated information sheet that detailed the intervention aim, timeline and components, and suggested strategies to promote their agents to sit less and move more at work. Support emails were also sent, which contained an infographic encouraging and suggesting ways for advisors to break up prolonged periods of sitting and be active during breaks. **Intrapersonal Strategies:** A 40-min researcher-led education and training sessions in week 1 and 5. Sessions introduced (week 1) and reinforced (week 5) the benefits of moving more and sitting less each day at work and the risks of prolonged sitting and standing. Using the intervention components as a point of departure, agents engaged in guided discussions to identify how they could utilise each intervention component to facilitate their behaviour change. Agents were given the opportunity to discuss their intervention experiences, including barriers to sitting less and moving more. In week 1 agents wrote a short-term goal to help them sit less and move more at work, for example, ‘I will go for a walk during my lunch break tomorrow’. This goal was discussed and reflected on in the week 5 session. |

| | | |
|---|---|---|
| Pickens et al (2016) **[17]** | Stand-capable desks | Employees received brief training on their new workstations. The workstations used for this intervention were the SteelCase™ (Grand Rapids, MI) Series 5 Desk (sit-to-stand) which were adjustable with an electronic motor from 65 cm to 130 cm, allowing the user to adjust the desk surface height for both sitting and standing. In comparison to a sit-to-stand workstation that can be adjusted by the user to any posture between seated and standing during the day, the stand-biased workstation is adjusted to a range of standing heights. The stand-biased group used the same workstations but were used only in individually set height ranges relative to the floor. The sit-to-stand group used a SteelCase™ Think Chair Model 6205 that had an adjustable seat height ranging from 40.5 cm to 53 cm and most were paired with anti-fatigue mats from Uline® (model H-2011). Stand-biased subjects used the Neutral Posture Inc. mesh back stool (seat height: 64.5 cm and 91.5 cm) with an attached footrest platform. Most stand-biased workstations (83%) were also equipped with an additional Wall-Saver footrest from Neutral Posture Inc. for under the desk. Monitor arms from Neutral Posture Inc. were purchased and installed for both types of stand-capable workstations for a dual monitor setup. |
| Rempel et al (2016) **[18]** | Forearm support band, trackball and ergonomics training | The arm board is a wraparound, padded arm support that attaches to the top, front edge of the work surface (30.5 cm depth, 76.2 cm width, 2.5 cm height: MorencyRest, R&D Ergonomics, Freeport, ME, USA). The trackball (16.5 cm depth, 8.6 cm width, 4.6 cm height, with a 4 cm diameter ball; Marble Mouse, Logitech, Fremont, CA, USA) was installed next to the keyboard. The ergonomics training involved conventional recommendations: 15 which included maintaining an erect posture while sitting, adjusting the chair height so that the thighs were approximately parallel to the floor, adjusting the arm support and worksurface height so that the forearms were approximately parallel to the floor, adjusting the mouse and keyboard location to minimise the reach, adjusting the monitor height so that the centre of the monitor is approximately 15 degrees below the visual horizon, and a reminder to take scheduled breaks. |

| | | |
|---|---|---|
| Schneider et al (2012)<br>**[19]** | Biofeedback on Voice Use | To prevent occupational voice disorders, the vocal awareness needs to be trained. A biofeedback software program that monitors the main parameters F0, SPL, and syllables per minute has been developed and introduced into the workplace environment of CCA. This biofeedback tool can provide real-time biofeedback for the employee during conversation with a customer by phone or afterward as summarised feedback. |
| Sharifi, Denesh and Gholamnia (2022)<br>**[20]** | Multicomponent ergonomic intervention | 1. **Comprehensive office ergonomic training:** two 90-minute group training sessions were held at the workplace to increase participants awareness of basic office ergonomic principles. Topics included etiology of work-related musculoskeletal disorders (WRMSDs) and significance of work layout alterations and workplace stretching exercises to avoid WRMSD. Participants were also taught risk self-assessment skills so they could readjust the workplace accordingly. At the end of the session, a concise visual pamphlet, which consisted of all the materials taught during the sessions, was given out.<br>2. **Work layout improvement**: Modifications were made to the physical workstation, including improvements to existing chairs and replacement of non-adjustable chairs equipped with arm and head rests and provision of footrests and standard stands for the screens.<br>3. **Supervised on-site face to face visits**: The day after the training sessions, researchers monitored how the education was translated into the working day. These visits also utilised motivational interviewing to elevate intrinsic motivation. Snapshots were taken if there was an inappropriate exercise. Pictures were put into a discussion and potential solutions were generated. After face-to-face training researchers visited participants on a bimonthly basis to ensure consistent healthy work practices and postural habits.<br>4. **Provision of quality break time encompassing regular exercise program**: Employees were given an additional rest break opportunity halfway through their shift. It was up to employees to select this time (that did not impact the workflow). During the training program the length of the session was discussed, and 10 minutes was agreed. Participants were advised to perform a set of stretching and joint mobilization exercises, targeting the whole body, once a day when they felt their muscles were tense or fatigued. A log was then given to each participant to be ticked everyday when the exercise had been |

| | | |
|---|---|---|
| | | performed. Later it was then monitored by supervisors and researchers on a weekly and bimonthly basis, respectively. |
| Tham (2004) **[21]** | Temperature and outdoor air supply | Blind intervention settings of temperature (T) and ventilation (V). Set points: T1 =22.5C, T2 =24.5C, V1=51 /s/p, V2=101 /s/p.<br><br>**Transitions**: Week 1 (T1+V1), week 2 (T2+V1), week 3 (T1+V1), week 4 (T1+V2), week 5 (T2+V2), week 6 (T2+V1), week 7 (T2+V2), week 8 (T1+V2), Week 9 (T1+V1). |
| Thatcher et al (2020) **[22]** | Office Plants: Working environment *(study 1)* | A total of 21 large, 40 cm grow pots and 3 large, 60 cm rectangular pots were installed in the office space amounting to one plant unit for about every 14 m2. The foliage plants used were Sanserveria Trifasciata (mother-in-law tongue; 9 × 40 cm grow pots), Chamaedorea Seifritzii (reed palm; 8 × 40 cm grow pots), Ficus Alii (banana-leaf fig, 3 × 40 cm grow pots), Ficus Lyrata (fiddle leaf fig, 1 × 40 cm grow pot), and Aglaonema (silver queen; 3 × 60 cm, rectangular pots). |
| | Office Plants: Working environment *(study 2)* | A total of 21 large, 40 cm grow pots and 3 large, 60 cm rectangular pots were installed in the office space amounting to one plant unit for about every 14 m2. The foliage plants used were Sanserveria Trifasciata (mother-in-law tongue; 9 × 40 cm grow pots), Chamaedorea Seifritzii (reed palm; 8 × 40 cm grow pots), Ficus Alii (banana-leaf fig, 3 × 40 cm grow pots), Ficus Lyrata (fiddle leaf fig, 1 × 40 cm grow pot), and Aglaonema (silver queen; 3 × 60 cm, rectangular pots). |
| Wargocki, Wyon and Fanger (2003) **[23]** | Air filters and outdoor air supply rates | 1. New versus used filter (a filter that had been in place for 6 months, the normal service life of the filter in this call-center).<br>2. Constant outdoor air supply rate 8% versus 80% of total supply air flow.<br><br>All combinations of the two interventions new/used filter, low/high outdoor air supply rate occurred in each of two successive 4-week periods, each combination being maintained for one week at a time. |

| | | |
|---|---|---|
| | | Week 0 (used filter & low outdoor air supply), week 1 (new filter & low outdoor air supply), week 2 (new filter & high outdoor air supply), week 3 (used filter & high outdoor air supply), week 4 (used filter & low outdoor air supply), week 5 (new filter & low outdoor air supply), week 6 (new filter & high outdoor air supply), week 7 (used filter & high outdoor air supply), week 8 (used filter & low outdoor air supply). |
| Workman & Bommer (2003) **[24]** | Alignment job design (AJD) | The alignment effort was to set performance measures congruent with business objectives inasmuch as what is measured becomes a goal or a milestone. A key aspect of the alignment would reduce the pressure to quickly solve problems so that specialists could focus on giving correct solutions. Management continued to track the number of problems solved, but only in the aggregate. The existing management structure was kept in place, and managers continued to conduct performance reviews. Performance rewards, however, such as bonuses, raises, and expressions of management approval, were administered based upon the new measures. This intervention also included a new process to support the strategic goals and facilitate learning. Coined the "hot seat," the process was devised to enable specialists to spend some portion of their time off the phones and working on problems in their open-problem queues. Support specialists would work three days on the phone (hot seat) and two days off the phone working on problems they had been unable to solve. |
| | High-involvement work processes (HIWP) | Structural changes - (1) elevating member–leader participation, (2) establishing customer and business feedback loops, (3) expanding member knowledge of the total work system, and (4) creating structural alignment.<br>This was accomplished by instituting member-leader cross participation process-improvement teams (PITs) for ongoing job-redesign efforts. Among the structural changes devised by the PITs was a formal escalation team, into which specialists would rotate off of the phones on a biweekly basis. In the escalation team, junior specialists were paired with senior specialists who acted as mentors.<br>To encourage leader-member participation in PITs, managers and specialists attended a one-week training seminar that encouraged a participative environment and focused on achieving personal and organizational potential. In addition, a series of "lunch and learn participation workshops" were conducted with managers and support specialists at regular intervals. The lunch and learn sessions were also used |

| | | |
|---|---|---|
| | | to uncover stumbling blocks in the structure and processes, as well as to expand the specialists' knowledge of the larger work system by exposing them to companywide processes. Customer survey scores (good and bad) were discussed to increase customer issue awareness and to formulate team plans for corrective actions. Similar to the AJD-group, PITs in the HIWP-group restructured the performance measurement system to align it with strategic business objectives. Specifically, individual quotas were eliminated and replaced with quotas at the team level; and rather than percentage of volume, a percentage of problems were incorporated in open queues to drive down the number of difficult problems going unresolved. |
| | Autonomous work teams (AWT) | The teams were **group-focused** and self-managed; they collaborated on task assignments; they planned and scheduled their work; and they used group decision making. Team members developed written agreements covering peer review criteria and the roles and tasks the team would assume. To encourage participation in the teams, specialists attended the same type of training seminar as in the HIWP intervention. In the AWT intervention, the previous measurement and reward structure that concentrated on individual production was replaced with team-based measurements and rewards. The new structure focused on thorough problem research and resolution, and problems were worked as a collective in the teams. This group also created a work design similar to the hot seat used in the AJD group. On a one-day-a-week rotation, two members from each of the teams would man the phones, while unsolved problems were passed to those members in the teams who were off the phones. These teams also organized themselves into "specialties." For instance, team 1 tended to focus on problems involving networking, whereas team 2 tended to focus on problems involving core system components. Team members were allowed to self-select their specialty. |
| Workman & Bommer (2004) **[25]** | Alignment job design (AJD) | Efforts are directed at measurement and reward structures to align organisational and individual goals. **Employee involvement is low**, and the traditional management structure remains in place. The focus is on management driving the alignment of measurement and rewards. Three objectives were set for the AJD group intervention: Examine the performance measurements and determine their outcomes; adjust them according to strategic organizational objectives; and adapt the structure and reward systems around these new measures. During the examination phase, they found that as problem volumes continued to increase, |

| | | |
|---|---|---|
| | | specialists reached a point where they were forced to concentrate on simple problems and set the harder problems aside in their open problem queues in hopes of returning to them later. This had the effect of causing the most difficult problems to go unsolved for extended periods of time. Therefore, a key aspect of the alignment reduced the pressure to quickly solve problems so that specialists could focus on giving correct solutions - adjusting the measurement system toward thorough problem resolution (e.g., looking at the number of repetitive calls and eliminating quotas); further, the number of escalations was tracked, as was the number of problems in open problem queues. Performance reward, such as bonuses, raises and the expressions of management approval were administered based on the new measured. Job rotation was also implemented to allow advisors to work days off the phone to resolve their open problems. |
| | High-involvement work processes (HIWP) | Structural changes - (1) elevating member–leader participation, (2) establishing customer and business feedback loops, (3) expanding member knowledge of the total work system, and (4) creating structural alignment.<br><br>HIWP **raises individual discretion and involvement in the development of organisational structures** through team-oriented practices, problem-solving groups, or quality circles (member–leader cross-participation process improvement teams (PITs) were devised for ongoing job redesign efforts. The structural changes devised by the PITs included a formal escalation team (research team), into which specialists would rotate off the phones on a biweekly basis. In the research team, junior specialists were paired with senior specialists who acted as mentors, but it leaves the supervisory structure in place. To encourage leader–member participation managers and specialists attended a 1-week training seminar that encouraged a participative environment and focused on achieving personal and organizational potential. In addition, a series of 'lunch and learn participation workshops' were conducted with managers and support specialists at regular intervals. The lunch and learn sessions were also used to uncover stumbling blocks in the structure and processes, as well as to expose the specialists to company-wide processes to expand their knowledge of the larger work system. Employee involvement is moderate (problem-solving teams and quality circles), specialists are involved in important decisions. |

| | | |
|---|---|---|
| | Autonomous work teams (AWT) | **Entirely group focused**. AWT utilise group-level autonomy and redistribute the control of the group structure and processes to the group members, replacing traditional management with a cooperative of independent peers. Enables expertise to be shared.<br><br>The team (1) assigned jobs to members, (2) planned and scheduled work, (3) made service-related decisions, and (4) took action to remedy problems. Management relinquished control of performance measurement and assessment to the group. Team members developed written agreements covering roles and tasks the team would assume, along with peer review criteria. To encourage participation in the teams, specialists attended the same type of 1-week training seminar as the HIWP intervention.<br><br>The previous measurement and reward structure concentrating on individual production was replaced with team-based measurements and rewards. The new measurement structure focused on thorough problem research and resolution, and problems were worked as a collective in the teams. The teams in this intervention created a work design similar to the AJD group. On a 1-day weekly rotation, two members from each team would 'man the phones,' while unsolved problems were passed to those members in the teams who were 'off the phones.' These teams also organized themselves into 'specialties.' For instance, team 1 focused more on problems involving networking, whereas team 2 tended to focus on problems involving core system components. Team members selected their own specialty. Merit increases were given to each team as a whole based on meeting team-defined quality and productivity objectives. |
| Yesilyurt & Yelken (2020) **[26]** | Voice therapy | For voice therapy, vocal hygiene and diaphragm breathing training were given in two groups, but voice exercises and laryngeal massage were applied to each client in the form of a specific therapy program. Voice therapy was performed 1 time per week. Each session lasted 35-40 minutes and a total therapy period of 4 weeks. Within the scope of vocal hygiene training, the clients were informed about different forms of vocal rest, suggestions to prevent reflux, not speaking in noisy places, resting the voice occasionally while talking, avoiding extreme behaviours |

| | | |
|---|---|---|
| | | related to phonation, speaking at the middle pitch and violence level, increasing hydration, avoiding substances-foods and drinks that could harm the sound. |
