# Supplementary material for: A systematic scoping review of health-promoting interventions for contact centre employees examined through a behaviour change wheel lens
Source: PLoS One. 2024 Mar 8;19(3):e0298150. doi: 10.1371/journal.pone.0298150 (PMC10923409; doi:10.1371/journal.pone.0298150)
Supplement: S2 Table — (PDF) [file pone.0298150.s006.pdf]

S2 Table - Characteristics Table for the 28 Included Intervention Studies

Characteristics of the 28 included intervention studies (from 26 intervention articles)									
**Study Details**			**Intervention Details**						
**Authors (Year); Country; Contact centre details***	**Population**	**Design****	**Duration**	**Data collection time point(s)**	**Intervention delivery (setting)*****	**Theoretical underpinning**	**Intervention aim(s)**	**Primary outcome(s)**	**Other outcome(s)**
**Allexandre et al (2016); USA; Corporate call centre [1]**	- Contact centre advisors<br>**- N**=161; 83.2% **female**<br>**- Mean age**: 40 years (SD 13)<br>- 77% **C/W**	RCT	8 weeks	Baseline, 8 and 16 weeks, and 1-year	**I1 (hybrid):** Ed/T/P (weekly mindfulness themes and meditation techniques taught through written and audio formats; educational articles (e.g., mindfulness in everyday eating) and x2 email reminders).<br>**I2 (office for meetings; hybrid for I1):** Ed/T/P (as I1) and En (followed by face-to-face group meetings, 1h x1 per week x 8 weeks; discussion and sharing positive experiences).<br>*- Delivered by selected company employees who participated in the web-based programme before the start of the study*.<br>**I3 (office for meetings; hybrid for I1):** Ed/T/P (as I1) and En (Face-to-face group meeting, discussion facilitated by clinical support on week 3, 6 and 8.)<br>*- Delivered by a clinical counsellor or licensed social worker*).<br>**C (office):** Waitlist control filling in outcome questionnaires only.	Based on mindfulness meditation principles	Increase mindfulness to reduce workplace stress	Perceived psychosocial stress	- Mindfulness<br>- Burnout<br>- Psychological and emotional wellbeing<br>- Productivity<br>- Acceptability and feasibility of the intervention
**Bond, Flaxman & Blunce (2008); UK; Two contact centres of a large financial services organization [2]**	- Contact centre advisors<br>**- N**=181; 67% **female**<br>**- Mean age**: 33 years (SD 10)<br>**- Ethnicity** NA	cRCT	14 months	2 months pre-intervention and 14 months	**I (office):** En/ER (steering group formed x12 team members; identified work organization problem areas and implemented change; including increased job control and meetings with team leaders to solve problems and develop training needs).<br>*- Delivered as a collaborative relationship between the researchers and organization members.*<br>**C (office):** Work as usual.	Based upon the principles of participative action research	Increase job control	Job control (moderated by psychological flexibility)	- Psychological distress<br>- Absence levels<br>- Intrinsic job motivation
**Chau et al (2015); Australia; Large telecommunication s company [3]**	- Contact centre advisors<br>**- N**=31; 45% **female**	Q-E	19 weeks	Baseline, 1, 4, and 19 weeks. Measurements conducted across the whole week	**I (office):** ER/T/P (sit-stand desk; brief training; daily e-mail reminders to stand for the first 2 weeks post installation).<br>*- Deliverer unclear.*<br>**C (office):** Work as usual with regular desks.	-	Reduce sitting time and increase physical activity	- Work and non-work sitting<br>- Work and non-work physical activity	- Productivity

	**- Mean age**: 33 years (SD 10.8)<br>**- Ethnicity** NA			(Monday-Saturday).					
**Chi & Lin (2009); Taiwan, China; Established under the Employment and Vocational Training Administration [4]**	- Contact centre advisors (with disability)<br>- **N**=22; 50% **female**<br>**- Mean age**: 25.6 years (range 24-55)<br>**- Ethnicity** NA	PP	12 months	2.5, 5 and 12 months	**I (office):** ER (screen filter fitted).<br>*- Deliverer unclear.*	-	Reduce visual fatigue	Visual fatigue	-
**Cook et al (2004); Australia; Newspaper contact centre [5]**	- Contact centre advisors<br>- **N**=59; 91.5% **female**<br>**- Mean age**: 39 years (range 21-68)<br>**- Ethnicity** NA	RCT	6 weeks	1, 6 and 12 weeks	**I (office):** ER/T (workstations adjusted to provide forearm support; prompt sheet given on how to maintain forearm support).<br>*- Delivered by researchers.*<br>**C (office):** Desks as usual, then given the intervention at 6 weeks.	-	Decrease neck/shoulder and wrist/hand musculoskeletal discomfort	Musculoskeletal discomfort	- Workstation setup<br>- Working posture<br>- Comfort
**Garrett (2016); USA; Provide telephonic health and clinical advising [6]**	- Contact centre advisors<br>- **N**=167; 70.7% **female**<br>**- Mean age** NA<br>**- Ethnicity** NA	Q-E	6 months	Continuously over 6 months	**I (office):** T/ER (training, stand-capable workstations, dual monitor set up, footrests and anti-fatigue mats installed).<br>*- Deliverer unclear.*<br>**C (office):** Seated at a traditional desk with dual monitor set up.	-	Reduce sitting time and increase standing time at work, and increase productivity	- Sitting time at work<br>- Standing time at work<br>- Productivity	-
**Holman et al (2009); UK; Department in a large UK company providing health insurance and health care [7]**	- Contact centre advisors, administrative employees and support section employees<br>- **N**=119; 55% **female**<br>**- Mean age** NA<br>**- Ethnicity** NA	Q-E	6 months (to fully implement)	1-month pre intervention and 1-month after full implementation	**I (office):** En/ER/T (assessment and redesign *(phase one)* to identify obstacles; one day off site x3. Implementation *(phase two)* occurred over the next few months, with progress monitored by the team's representatives who attend progress meetings over 3 months. Design changes involved training to increase skills).<br>*- Delivered by the research team (facilitated phase one) and employees (led proposed initiatives).*<br>**C (office):** Serendipitous event created an inert-treatment control group (outsourcing	Job demands – resources theories of job design	Enhance job design characteristics	Job-related wellbeing	- Job control<br>- Decision making<br>- Feedback<br>- Skill utilisation<br>- Task obstacles

					initiative, announced and introduced by management just before the assessment and redesign phase. This meant the key active ingredient of the job redesign did not occur).				
**Holman & Axtell (2016); UK; Department in the UK civil service dealing with transport-related issues [8]**	- Contact centre advisors<br>**- N**=62; 56% **female**<br>**- Mean age** NA<br>**- Ethnicity** NA	Q-E	6 months (to fully implemen t)	1-month pre intervention and 2 months after full implementation	**I (office):** En/ER/T (assessment and redesign *(phase one)* to identify obstacles; two-day workshop then 2 weeks to develop proposals for a meeting with the managers and researchers. Implementation *(phase two)* occurred over the next few months, with the effectiveness monitored by the teams. Design changes involved training to increase job control).<br>- *Delivered by the research team (facilitated phase one) and employees (led proposed initiatives).*<br>**C (office):** Active control – completed initial survey.	Multiple mediator/mul tiple outcome model of job redesign intervention	Change job characteristics	Job-related wellbeing	- Job control and feedback<br>- Job performance<br>- Psychological contract fulfilment
**Kennedy & Pretorius (2008); South Africa; Assists external company's staff with mental health/behavioural problems [9]**	- Contact centre advisors<br>- N=19; 78.9% female<br>**- Mean age** NA<br>**- Ethnicity** NA	PP	NA	Baseline and end of the intervention	**I (office):** ER/Ed/P (portable heart rate variability biofeedback device; guides users by finding their unique breathing pattern so that respiration and heart rate are synchronised; points awarded for smooth waves).<br>- *Deliverer unclear.*	-	Reduce work-related stress symptoms	Stress-related symptoms	Environmental stressors (supportive work environment and resource management).
**Kirk et al (2013); Australia; Major metropolitan contact centre [10] *(Study 1)***	- Contact centre advisors<br>**- N**=214; **Female** NA<br>**- Mean age** NA<br>**- Ethnicity** NA	Q-E	NA	1-month pre- and post-intervention	**I (office):** ER/T (paper-based ergonomic checklist delivered in-situ to each team member).<br>- *Designed by the in-house occupational health and safety officer and team leaders delivered the checklist.*<br>**C (office):** Work as usual.	-	Reduce work-related musculoskelet al disorders	Physical discomfort	-
**Kirk et al (2013); Australia; Major metropolitan contact centre [10] *(Study 2)***	- Contact centre advisors<br>**- N**=NA; **Female** NA<br>**- Mean age** NA<br>**- Ethnicity** NA	Q-E	NA	1-month pre- and post-intervention	**I (office):** T/Ed (based on a paper-based ergonomic checklist; one-on-one skill-based training session included demonstration on adjustments and positions, followed by rehearsal; training followed by an explanation of the positive effects of the training and the health consequences of common errors).<br>- *Delivered in-situ by the researcher.*<br>**C (office):** Work as usual.	-	Reduce work-related musculoskelet al disorders	Physical discomfort	-

**Krajewski, Wieland & Sauerland (2010); Germany; Inbound contact centre [11]**	- Contact centre advisors<br>**- N**=14; 57% **female**<br>**- Mean age**: 38 years<br>**- Ethnicity** NA	RCT	6 months	1 week pre-intervention (baseline) and 2, 4, and 6 months. Measurements conducted at 12:00, 13:00, 16:00, and 20:00 on assessment days.	**I (office):** ER/T (lunch break with progressive muscle relaxation; snack served, then progressive muscle relaxation in the 'silent room', wear eye masks, lie on medical daybed, instructions through headphones with calming music).<br>*- Delivered by researchers.*<br>**C (office):** lunch break involving small talk in company staff room with self-chosen colleagues.	Systematic relaxation techniques	Reduce stress levels	Strain states (emotional, mental, motivational and physical)	-
**Krajewski, Sauerland & Rainer (2011); Germany; Contact centre details not available [12]**	- Contact centre advisors<br>**- N**=14; 57.1% **female**<br>**- Mean age:** 38.4 years<br>- 100% **C/W**	RCT	6 months	1-day per month at five time points (awakening, awakening +30 min, start of lunch break, end of lunch break, and bedtime)	**I (office):** ER/T (lunch break with progressive muscle relaxation; snack served, then progressive muscle relaxation in the 'silent room', wear eye masks, lie on medical daybed, instructions through headphones with calming music).<br>*- Delivered by researchers.*<br>**C (office):** lunch break involving small talk in company staff room with self-chosen colleagues.	Cognitive-behavioural model of relaxation	Reduce stress levels	Cortisol levels (through saliva samples) to indicate stress levels	-
**Lehto et al (2003); Finland; The largest Finnish telecommunications operator [13]**	- Contact centre advisors (without severe voice problems)<br>**- N**=48; 79% **female**<br>**- Mean age**: 27.5 years (range 21-40)<br>**- Ethnicity** NA	PP	2 days	Baseline and 3 weeks	**I (office):** T/Ed (vocal training on 2 days including vocal hygiene, activities and practice).<br>*- Delivered by a speech language therapist and speech teacher.*	-	Reduce voice failures	Perceived vocal symptoms	- Acceptability and feasibility of the intervention
**Mishra et al (2010); India; Four different business process outsourcing companies [14]**	- 23.1% managers/ admin staff, 74.2% advisors and 2.8% researchers/ analysts who smoked<br>**N**= 646; 19.7% **female**	cRCT	12 months	Across 18 months: regular follow up visits every 2-3 months	**I1 (unclear):** Ed/En/P (educational health awareness sessions followed by focus group discussion to reflect on barriers to smoking cessation and to identify strengths to overcome these (7-10 employees).<br>**I2 (unclear):** Ed/En/P (I1 above plus one-to-one counselling was provided to address rationalizations for continuing tobacco use at an individual level)<br>**I3 (unclear):** Ed/En/P (I2 above plus bupropion was offered based on individual need assessment)	-	Increase tobacco cessation	Tobacco cessation	Knowledge, attitude and practice regarding tobacco use

	**- Mean age**: 23.1 years (SD 3.7)<br>**- Ethnicity** NA				*- Delivered by researchers (sessions) and expert tobacco counsellor (focus groups and one-to-one counselling).*<br>**C (unclear):** Distribution of pamphlets (information on hazards of tobacco).				
**Morris et al (2021); UK; Inbound contact centre [15]**	- Contact centre advisors<br>- **N**=59; 68% **female**<br>**- Mean age**: 30.6 years (SD 11.5)<br>**- Ethnicity** NA	RCT	10 months	Baseline, 3 and 10 months	**I1 (office):** En/T/M/P/Ed (x4 30-min education and training sessions; stand-up champions and team leader support; self-monitoring and health check feedback provided).<br>**I2 (office):** ER/En/T/M/P/Ed (multicomponent intervention with height-adjustable workstations; instructions provided and reminders to stand via emails; x4 30-min education and training sessions; stand-up champions and team leader support; self-monitoring and health check feedback provided).<br>*- Delivered by researchers.*	Socioecological model, the COM-B model and Behaviour Change Wheel	Reduce sitting time and increase standing and movement time at work	Sitting time at work	- Feasibility and acceptability of the intervention<br>Various outcomes for:<br>- Anthropometric<br>- Behavioural<br>- Cardiometabolic<br>- Musculoskeletal<br>- Psychosocial (wellbeing)<br>- Work
**Morris et al (2019); UK; Inbound contact centre [16]**	- Contact centre advisors and team leaders<br>- **N**=19; 78% **female**<br>**- Mean age**: 39.3 years (SD 11.9)<br>- 78% **C/W**	PP	8 weeks	Baseline and 8 weeks	**I (office):** ER/En/T/P/Ed (multicomponent intervention with height adjustable workstation; team leaders provided daily verbal support in one-to-one meetings; 1-hour education and training session week 1 and 5; weekly emails).<br>*- Delivered by researchers.*	Socioecological model, the COM-B model and Behaviour Change Wheel	Reduce sitting time and increase standing and movement time at work	Acceptability and feasibility outcomes	Various outcomes for:<br>- Behavioural<br>- Cardiometabolic<br>- Anthropometric
**Pickens et al (2016); USA; Provided telephonic health and clinical advising [17]**	- Contact centre advisors<br>**- N=138**; 66.7% **female**<br>**- Mean age:** 32.9 years<br>- 22.5% **AA**, 70.3% **C/W**	Q-E	6 months	A 2-day collection period at baseline, 3 and 6 months	**I1 (office):** T/ER (training; sit-to-stand workstation, dual monitor set up, anti-fatigue mats and chair with adjustable seat hight - purchased by the company after consultation with the research team).<br>**I2 (office):** T/ER (training/ stand-biased workstation, mesh back stool and footrest - purchased by company).<br>*- Delivered by managers (assigned and purchased desks to install).*<br>**C (office):** Seated at a traditional desk.	-	Reduce sitting time and increase standing time at work	- Sitting time at work<br>- Standing time at work	- Physical activity levels<br>- Workstation usage<br>- Acceptability and feasibility of the intervention
**Rempel et al (2006); USA; Large healthcare company [18]**	- Contact centre advisors<br>**- N=182**; 94% **female**	RCT	52 weeks	Weekly for 52 weeks	**I1 (office):** T/ER (ergonomic training; trackball installed).<br>**I2 (office):** T/ER (ergonomic training; armband provided).<br>**I3 (office):** T/ER (ergonomic training; trackball + armband).<br>*- Interventions delivered by researchers.*	-	Prevent upper body musculoskeletal disorders	Pain intensity (neck/shoulders, right elbow/forearm/wrist/hand, and left elbow/forear	- Work schedule<br>- Medication uses for pain<br>- Acute injury events during the week

	**- Mean age**: 40 years (SD 11.6)<br>- 47.8% **C/W**, 20.9% **AA**, 17.6% **A/P**, 1.1% **N-A**				**C (office):** Ergonomic training only.			m/ wrist/hand)	
**Schneider et al (2012); Austria; One of the largest Austrian telecommunication companies [19]**	- Contact centre advisors<br>**- N**=76; 49% **female**<br>**- Mean age**: 29.3 years<br>**- Ethnicity** NA	Q-E	4 weeks	Baseline and 4 weeks	**I1 (office):** ER/Ed (biofeedback programme installed; providing real time feedback).<br>*- Delivered by researchers.*<br>**C (office):** The control group used the biofeedback programme similarly, but the results were blinded and made visible only to the examiners.	-	Improve vocal health	- Vocal self-assessment<br>-Vocal performance	- Vocal constitution<br>- Vocal risk factors
**Sharifi, Denesh and Gholamnia (2022); Iran; Private telecommunication company [20]**	- Contact centre advisors<br>**- N**=84; 76% female<br>**- Mean age**: 28.1 years (SD 3.69)<br>**- Ethnicity** NA	PP	6 months	Baseline and 6 months	**I (office):** ER/T/Ed/E (Modifications to the physical workstation, and adjustable chairs with arm rests, footrests and screen stands added to the environment; given an additional 10-minute rest break to perform an exercise program; Ergonomic skills training; visual pamphlet on ergonomic skills training; educated on the etiology of MSD; snapshots of inappropriate exercises taken to discuss potential solutions; Log to track daily exercises; researchers monitored ergonomic behaviours and tracked exercise program participation).<br>*- Delivered by researchers.*	-	Reduce work-related musculoskeletal symptoms	- Musculoskeletal symptoms	- Fatigue<br>- Mental workload
**Tham (2004); Singapore; Telecommunication company [21]**	- Contact centre advisors<br>**- N**=56, 100% **female**<br>**- Mean age**: 28 years (range 25-36)<br>**- Ethnicity** NA	PP	9 weeks	Weekly (morning and afternoon)	**I1 (office):** ER **(**temperature 22.5C and 51 set points (level of outdoor air supply rates)).<br>**I2 (office):** ER **(**temperature 24.5C and 51 set points (level of outdoor air supply rates)).<br>**I2 (office):** ER **(**temperature 22.5C and 101 set points (level of outdoor air supply rates)).<br>**I2 (office):** ER **(**temperature 24.5C and 101 set points (level of outdoor air supply rates)).<br>*- Deliverer unclear.*	-	Reduce sick building syndrome symptoms	Sick building syndrome symptoms	- Perceptions of the indoor environmental conditions<br>- Self-assessed productivity
**Thatcher et al (2020); South Africa; Large IT company (clients in Africa, Europe, and the Middle East) [22]**<br>***(Study 1)***	- Contact centre advisors<br>**- N**=32; 56% **female**<br>**- Mean age**: 31.6 years (SD 10.8)	PP	6 weeks	Baseline and 6 weeks	**I (office):** ER (plant provision into the office).<br>*- Delivered by researchers.*	Attention Restoration Theory and Stress Reduction Theory	Improve wellbeing	Perceived psychological and physical wellbeing	- Perceived productivity<br>- Perceived work engagement<br>- Evaluations of the work environment<br>**-** Connectedness to nature

	**- Ethnicity** NA								
**Thatcher et al (2020); South Africa; Small medical insurance company [22]** ***(Study 2)***	- Contact centre advisors<br>**- N**=34; 56% **female**<br>**- Mean age**: 28.9 years (SD 4.7)<br>**- Ethnicity** NA	PP	14 weeks	Baseline and 14 weeks	**I (office):** ER (plant provision into the office).<br>*- Delivered by researchers.*	Attention Restoration Theory and Stress Reduction Theory	Improve performance and wellbeing	Perceived psychological and physical wellbeing	- Perceived productivity<br>- Absenteeism<br>- Perceived work engagement<br>- Job satisfaction.<br>- Evaluations of the work environment<br>- Perceived attractiveness of the plants
**Wargocki, Wyon and Fanger (2004); Denmark; Addressed national directory enquiries [23]**	- Contact centre advisors<br>**- N**=26; **Female** NA<br>**- Mean age**: NA<br>**- Ethnicity** NA	PP	Each intervention alternated weekly; each implemented twice across 8 weeks	1-week	**I1 (office):** ER (new filter with low outdoor air supply rate).<br>**I2 (office):** ER **(**new filter with high outdoor air supply rate).<br>**I2 (office):** ER **(**used filter with high outdoor air supply rate).<br>**I2 (office):** ER **(**used filter with low outdoor air supply rate).<br>*- Deliverer unclear.*	-	Reduce sick building syndrome symptom intensity	Sick building syndrome symptom intensity	- Perceived air quality (including acceptability measures)<br>- Environmental perceptions<br>- Self-estimated productivity
**Workman (2003); USA; Large international computer company [24]**	- Contact centre advisors<br>**- N=149**; 44% **female**<br>**- Mean age**: NA<br>**- Ethnicity** NA	RCT	NA	Baseline and post-intervention	**I1 (office):** ER/En/In/P (alignment job redesign; employee structure and reward system aligned to organizational goals e.g. management approval and raises).<br>**I2 (office):** ER/En/T/Ed/P (high-involvement work processes; raises individual discretion and involvement in organizational development; team problem solving; mentors assigned; 1-week training programme for advisors and managers; lunch and learn participation workshops to expand knowledge of the larger work system).<br>**I3 (office):** ER/En/T/In/P (autonomous work teams; entirely group focused redistributing control and creating written contracts for roles; team measures own performance; 1-week training seminar; team-based measurements and rewards).<br>*- Deliverer unclear.*<br>**C (office):** Work as usual.	Alignment theory and Cooptimized systems theory	Improve employee job attitudes	Job satisfaction	- Task relationship orientation
**Workman & Bommer (2004); USA; Large**	- Contact centre advisors	RCT	6 months	Baseline and 6 months post-intervention	**I1 (office):** ER/En/In/P (alignment job redesign; employee structure and reward	Alignment theory and	Improve employee job attitudes	Job satisfaction	- Organizational commitment

**international computer company [25]**	**- N=149**; 44% **female**<br>**- Mean age**: 31 years (range 21-56)<br>**- Ethnicity** NA				system aligned to organizational goals e.g. management approval and raises).<br>**I2 (office):** ER/En/T/Ed/P (high-involvement work processes; raises individual discretion and involvement in organizational development; team problem solving; mentors assigned; 1-week training programme for advisors and managers; lunch and learn participation workshops to expand knowledge of the larger work system).<br>**I3 (office):** ER/En/T/In/P (autonomous work teams; entirely group focused redistributing control and creating written contracts for roles; team measures own performance; 1-week training seminar; team-based measurements and rewards).<br>*- Delivered by external consultants (organizational developers).*<br>**C (office):** Work as usual.	Cooptimized systems theory			- Degree of group orientation
**Yesilyurt & Yelken (2020); Turkey; Contact centre details not available [26]**	- Contact centre advisors with voice problems<br>- **N**=13; 100% **female**<br>**- Mean age**: NA<br>**- Ethnicity** NA	PP	4 weeks	Baseline and 4 weeks	**I (unclear**): Ed/T (voice therapy, vocal hygiene and diaphragm breathing training given in groups; voice exercises and laryngeal massage delivered individually; all performed x1 per week lasting 35-40 minutes).<br>*- Deliverer unclear.*	-	Improve vocal health	Vocal health	-

Footnotes: NA: Not available; I: intervention; C: control; SD: Standard deviation; C/W: Caucasian/White; AA: African American; A/P: Asian or Pacific Islander; N-A: Native American.
*** See online supplementary 7 for intervention study reference list.**
**** Study designs: RCT: Randomised controlled trial, cRCT: Clustered randomised controlled trial, Q-E: Quasi-experimental, PP: Pre-post study (within-subjects).**
***** Intervention function: T: Training, En: Enablement, P: Persuasion, Ed: Education, ER: Environmental restructuring, In: Incentivisation, M: Modelling.**
