# Supplementary material for: A systematic scoping review of health-promoting interventions for contact centre employees examined through a behaviour change wheel lens
Source: PLoS One. 2024 Mar 8;19(3):e0298150. doi: 10.1371/journal.pone.0298150 (PMC10923409; doi:10.1371/journal.pone.0298150)
Supplement: S3 Table — (PDF) [file pone.0298150.s007.pdf]

S3 Table: Risk of Bias assessment tables

Risk of bias assessment for interventions with a quasi-experimental design							
**Study**	**Confounding**	**Selection of participants**	**Classification of interventions**	**Deviations from intended interventions**	**Missing outcome data**	**Measurement of the outcome**	**Selection of the reported results**
**Chau et al (2015) [58]**	Low	Low	Low	Some concerns	Low	Low	Low
**Garrett (2016) [53]**	High	Low	Low	Low	Low	Low	Low
**Holman et al (2009) [48]**	High	Low	Low	Low	Low	Some concerns	Low
**Holman & Axtell (2016) [60]**	Low	Low	Low	Some concerns	Low	Low	Low
**Kirk et al (2013) – Study 1 [41]**	High	Low	High	No information	No information	Some concerns	Low
**Kirk et al (2013) – Study 1 [41]**	High	Low	High	No information	No information	Some concerns	Low
**Pickens et al (2016) [62]**	Low	Low	Low	Some concerns	Low	Some concerns	Low
**Schneider et al (2012) [63]**	High	Low	Low	Low	Low	Low	Low

Risk of bias assessment for interventions with a randomised or cluster-randomised-controlled design						
**Study**	**Randomisation process**	**Deviation from intended interventions (assignment)**	**Deviation from intended interventions (adherence)**	**Missing outcome data**	**Measurement of the outcome**	**Selection of the reported results**
**Allexandre et al (2016) [49]**	Low	Low	High	Low	Some concerns	Low
**Cook et al (2004) [59]**	Low	Low	Low	Low	Some concerns	Low
**Krajewsji, Wieland & Sauerland (2010) [42]**	Low	Low	High	Low	Some concerns	Low
**Krajewski, Sauerland & Rainer (2011) [43]**	Low	Low	Low	Low	Low	Low
**Morris et al (2021) [10]**	Low	Low	Low	Low	Low	Low
**Rempel et al (2006) [50]**	Low	Low	Low	Low	Some concerns	Low
**Workman & Bommer (2004) [64]**	Low	Low	Low	Low	Low	Low
**Mishra et al (2010) [44]**	Low	Some concerns	Low	Low	Low	Some concerns
**Bond et al (2008) [57]**	Low	Low	Low	Low	Low	Low
**Workman (2003) [55]**	Some concerns	Low	Low	Low	Some concerns	Low

Risk of bias assessment for interventions with a pre-post study design													
**Study**	Study question	Eligibility criteria and study population	Study participants representative of clinical populations of interest	All eligible participants enrolled	Sample size	Intervention clearly described	Outcome measures clearly described, valid, and reliable	Blinding of outcome assessors	**Follow-up rate**	**Statistical analysis**	Multiple outcome measures	Group-level interventions and individual-level outcome efforts	Overall
**Chi & Lin (2009) [4]**	Yes	Yes	No	Not reported	Not reported	Yes	Yes	Not applicable	Yes	Yes	Yes	Not applicable	Fair
**Kennedy & Pretorius (2008) [9]**	Yes	Yes	Yes	Not reported	Not reported	Yes	No	Not applicable	Yes	Yes	Yes	Not applicable	Fair
**Lehto et al (2003) [13]**	Yes	Yes	Yes	Not reported	Not reported	Yes	No	Not applicable	Yes	Yes	No	Not applicable	Fair
**Sharifi, Denesh and Gholamnia (2022) [20]**	Yes	Yes	No	Yes	Not reported	Yes	Yes	Not applicable	Yes	Yes	No	Not applicable	Fair
**Tham (2004) [21]**	Yes	No	No	Not reported	Not reported	Yes	No	Not applicable	Not reported	Yes	Yes	Yes	Poor
**Thatcher at al (2020) study 1 [22]**	Yes	Yes	No	Yes	Yes	Yes	Yes	Not applicable	No	Yes	No	Yes	Fair
**Thatcher at al (2020) study 2 [22]**	Yes	Yes	No	Yes	Yes	Yes	No	Not applicable	No	Yes	No	Yes	Poor

**Wargocki, Wyon and Fanger (2004) [23]**	Yes	No	Not reported	Not reported	Not reported	Yes	No	Not applicable	Can’t determine	Yes	Yes	Yes	Poor
**Yesilyurt & Yelken (2020) [26]**	Yes	Yes	No	Yes	Not reported	Yes	Yes	Not applicable	Yes	Yes	No	Not applicable	Fair
