# Supplementary material for: A systematic scoping review of health-promoting interventions for contact centre employees examined through a behaviour change wheel lens
Source: PLoS One. 2024 Mar 8;19(3):e0298150. doi: 10.1371/journal.pone.0298150 (PMC10923409; doi:10.1371/journal.pone.0298150)
Supplement: Attachment — Submitted filename: Response to Reviewers.docx [file pone.0298150.s008.docx]

| **Reviewer 1** | **Authors Response** |
| --- | --- |
| **Line 40**: For the benefit of the reader, please briefly describe what do you mean by the term attrition. Also, figures for contact centre employees are compared with UK averages. Please briefly say who these averages relate to? (e.g. office based employees etc?). | Thank you for your comment. We have added a brief definition of attrition (line 46).  We have also made it clear that this attrition rate is being compared to the UK average rate across all industries (line 47). To the best of our knowledge no such attrition rate is available for office-based jobs. |
| **Line 75**: Can authors justify why they excluded non-English language studies at the eligibility stage especially when they mention that there was a low number of studies from low-and-middle income countries (LMICs)? Including non-English language studies at an earlier stage would have given an idea of how many were actually there and if required (because of issue of translating them into English or non-expertise in other languages), then they may have been excluded at a later stage. Including only studies published in English should be added as a limitation of the review. | We have acknowledged this as a limitation of the review. To address this, we returned to the screening of included studies, allowing us to identify three excluded studies not published in English. From this we could determine that none of these studies would have been included for additional reasons (e.g. not an intervention). We have explained this within the manuscript within the limitations (line 398-401). |
| **Line 92**: Authors mention extracting data on acceptability of the intervention. It would also be beneficial to know how acceptability was explored in the included studies (using qualitative, mixed methods?), and whether the authors considered or not considered a mixed methods review instead of just including study author's conclusion as evidence of acceptability and/or feasibility, and the reasons for doing so. | We have added a sentence (line 100-102) to explain how acceptability was reported through qualitative methods through participant feedback.  We reviewed acceptability/feasibility based on data extracted from the methods and results of the relevant intervention papers. We feel this does not need explaining within the manuscript. |
| **Line 98**: If quality of included studies was assessed then I would encourage authors to look into assessing quality of pre-post study designs as well. Examples of some tools that can be considered are NIH study quality assessment tool for before-after (pre-post) studies with no control group ([https://www.nhlbi.nih.gov/health-topics/study-quality-assessment-tools](https://urldefense.com/v3/__https:/www.nhlbi.nih.gov/health-topics/study-quality-assessment-tools__;!!IhKztkE!ciiiKqvloGTOMzUQr0q7RTYOZlKcgQlu16E8lKowfZWcX0WJpYWl-8Lfk6Zayyg2Px0KEYz-GXhmsOL2lcvN$)) or Evidence project risk of bias tool (Kennedy, C.E., Fonner, V.A., Armstrong, K.A. et al. The Evidence Project risk of bias tool: assessing study rigor for both randomized and non-randomized intervention studies. Syst Rev 8, 3 (2019). [https://doi.org/10.1186/s13643-018-0925-0](https://urldefense.com/v3/__https:/doi.org/10.1186/s13643-018-0925-0__;!!IhKztkE!ciiiKqvloGTOMzUQr0q7RTYOZlKcgQlu16E8lKowfZWcX0WJpYWl-8Lfk6Zayyg2Px0KEYz-GXhmsCm9i6xm$)). | Thank you for identifying these relevant tools. From this we decided to use the NIH tool.  We have added the pre-post quality assessment table into S6 Table, included the tool in the methods section and added an explanation as to why one of the pre-post studies was not quality assessed (Line 108-109), and included a summary of pre-post studies overall quality within the results (line 188-189). |
| **Line 182**: Can authors also say a little about interventions mapping to multiple domains. | We have included a sentence to explain what is meant by studies mapping to multiple domains, and why the primary intended outcome was used to categorise interventions (line 198-200). |
| **Line 217**: Please briefly elaborate on the term 'vocal hygiene' | Included explanation presented by the original study on line 235. |
| **Line 231**: "For the used-to-new air filter, participant acceptability of air quality decreased" can this sentence be rephrased, this is not very clear. | We have rephrased this, referring to the original study, to make the findings clearer (line 248-249). |
| **Line 316**: Briefly say why that is so (e.g. because no stated theory does not mean the intervention is not logical/sensible). | We have added a sentence to acknowledge that theory can be valuable but does not always predict/ensure the effectiveness of an intervention (339-341). |
| **Reviewer 2** | **Authors Response** |
| **L36** – is deprived the correct term? or is it lower socioeconomic background(s)? | We have now removed the reference to deprivation from the text (line 42) after exploring the literature further. |
| **L40** – are the UK averages comparable to similar job types (ie desk-based workers) or all industries? | Similar to reviewer 1’s comment, we have made it clearer that this attrition rate is being compared to the UK average rate across all industries (line 46). To the best of our knowledge no such attrition rate is available for office-based jobs. |
| **L102** – suggest using BCT throughout, rather than behaviour change taxonomies, and please add reference. | We have used the term ‘BCT’ throughout after the first use of the phrase ‘behaviour change techniques’, and added the reference (36) on line 119. |
| **L108** – should this read …had ‘completed’ BCT training… ? | Yes, thank you for spotting this. We have now addressed this (line 120). |
| **L126-143 –** this might work better in tabulated format. | The information in the text summarises the data provided in the characteristics table within supplementary 5 (line 139). We feel this level of detail is appropriate within the manuscript. |
| **L167-175** – again, tabulate? | The information in the text summarises the data provided in the risk of bias tables within supplementary 6 (line 182). We feel this level of detail is appropriate within the manuscript. |
| **L282** – where it says ‘relatively young’ it would be useful to quantify what this means. | We have included the mean age (32.5 years) to indicate what is meant by ‘relatively young’ (line 303). |
| **L310-311** – could you please add some discussion points to explain why coercion and restriction would be of interest to future research. | On reflection, a more appropriate discussion point for this relates to the inappropriate use of coercion and restriction within the workplace. We have added this on line 332-333. |
| **Discussion section** – i) RQ2 and 3 read like a summary of the findings and would benefit from the addition of discussion points. ii) It would be useful for the reader to have all the RQs repeated as the headers in the discussion section – I felt scrolling back to remind myself what they were was disruptive to reading. iii) Should there also be some commentary for RQ4? | i) We have now added a discussion point for RQ2 (363-365) and RQ3 (381-382).  ii) We have added the research questions into the headers of each discussion (line 289, 357 and 376).  iii) We have discussed RQ4 (evidence gaps requiring future research) throughout the discussion, and the same approach was taken for the results section (stated in line 124). |
| **Additional changes** | |
| **Line 35-36** | Having checked the references, the link for the webpage for reference one was broken. We have therefore used a new source, changing the statistic stated on line 35. |
| **Formatting** | We have edited the manuscript to meet PLOS ONE’s style requirements, including those for file naming, the headings within the manuscript, the reference list, and the supporting information files. |
